# *N*-Acetylcysteine combined with prednisolone treatment shows better hearing outcome than treatment with prednisolone alone for patients with idiopathic sudden sensorineural hearing loss: a retrospective observational study

**DOI:** 10.1007/s00405-023-08097-4

**Published:** 2023-07-01

**Authors:** Mussab Kouka, Nils Bevern, Julia Bitter, Orlando Guntinas-Lichius

**Affiliations:** Department of Otorhinolaryngology, Jena University Hospital, Am Klinikum 1, 07747 Jena, Germany

**Keywords:** Idiopathic sudden sensorineural hearing loss, Hearing gain, *N*-Acetylcysteine, Siegel classification, Japan classification

## Abstract

**Objectives:**

Internationally, corticosteroids are still the mainstay treatment for patients with idiopathic sudden sensorineural hearing loss (ISSHL). This is a retrospective monocentric study investing the impact of adding *N*-acetylcysteine (NAC) to prednisolone treatment on patients with ISSHL at a tertiary university otorhinolaryngology department.

**Methods:**

793 patients (median age 60 years; 50.9% women) with a new diagnosis of ISSHL from 2009 to 2015 were included in the study. 663 patients received NAC administration in addition to standard tapered prednisolone treatment. Univariate and multivariable analysis were performed to identify independent factors regarding negative prognosis of hearing recovery.

**Results:**

Mean initial ISSHL and hearing gain after treatment in 10-tone pure tone audiometry (PTA) were 54.8 ± 34.5 dB and 15.2 ± 21.2 dB, respectively. In univariate analysis, treatment with prednisolone and NAC was associated with a positive prognosis of hearing recovery in the Japan classification in 10-tone PTA. In multivariable analysis on Japan classification in 10-tone PTA including all significant factors from univariate analysis, negative prognosis of hearing recovery were age > median (odds ratio [OR] 1.648; 95% confidence interval [CI] 1.139–2.385; p = 0.008), diseased opposite ear (OR 3.049; CI 2.157–4.310; p < 0.001), pantonal ISSHL (OR 1.891; CI 1.309–2.732; p = 0.001) and prednisolone alone without NAC treatment (OR 1.862; CI 1.200–2.887; p = 0.005).

**Conclusions:**

Prednisolone treatment combined with NAC resulted in better hearing outcomes in patients with ISSHL than treatment without NAC.

## Introduction

An idiopathic sudden sensorineural hearing loss (ISSHL) is a sudden onset, usually unilateral, cochlear sensorineural hearing loss of ≥ 30 dB within < 3 days in at least 3 contiguous frequencies without an identifiable cause. The incidence is estimated to be between 8–400/100,000 cases [1–4]. Because of the unexplained cause, many therapies have been tried. Antivirals, thrombolytics, vasodilators, and rheologics seem to have no effect [5]. Internationally, there is no standard treatment for patients with ISSHL but current therapeutic approaches are mainly focused on different forms of application of corticosteroids. If there is no improvement in hearing after systemic corticosteroid therapy, local intratympanic application may be used. Recent studies are mainly concerned with the combination of local and systemic corticosteroid administration in first-line therapy. The aim of this study was to evaluate the adding administration of *N*-acetylcysteine (NAC) to prednisolone treatment on patients with ISSHL at a tertiary university otorhinolaryngology department.

NAC has several effects that are thought to be beneficial to cell stress in the inner ear [6]. Oxygenated radicals can damage hair cells in the inner ear by activating apoptotic cell death programs. NAC acts as a free radical scavenger and can decrease the cell's nitric oxide production by increasing the synthesis of reduced glutathione [7], thus decreasing the production of harmful nitrogen radicals [8]. In addition, NAC can prevent cell apoptosis as a donor of reduced glutathione [6]. In contrast to treatment for ISSHL, NAC is already a component of treatment for acute hearing loss of other etiologies. One application of NAC is hearing loss caused by aminoglycosides, which are used in tuberculosis treatment. Kranzer et al. reported of the convincing otoprotective effect of NAC in preventing aminoglycoside induced ototoxicity while tuberculosis treatment [9]. In literature, some studies exist on the therapeutic outcome of NAC in combination with steroid treatment in ISSHL, but the data is limited and inconclusive [10–13].

For this purpose, the impact of administration of NAC to prednisolone treatment of 793 patients with ISSHL who were hospitalized at a department of otorhinolaryngology in a tertiary university center in the period from 2009 to 2015 were analyzed.

## Methods

### Ethical considerations

This retrospective study was approved by the Ethics Committee of the BLINDED (IRB No. 4755-0416). The Ethics Committee waived the requirement for informed consent of the patients because the study had a non-interventional retrospective design and all data were analyzed anonymously.

### Patients

For this purpose, 920 patients were screened which were treated in the Department of Otorhinolaryngology, BLINDED, Germany, from September 2009 to December 2015. The patients were all coded according to the International Statistical Classification of Diseases and Related Health Problems, 10th revision (ICD-10) with the number H91.2 (ISSHL including acute hearing loss without further specification) [14]. The exclusion criteria were: Varicella-zoster virus infection, Herpes simplex virus 1/2 infection, toxic inner ear or acute otitis media, only one or no audiogram available, strong suspicion of aggravation, sarcoidosis, discontinuation of treatment, acute exacerbation of chronic otitis media, vestibular schwannoma, acute sepsis, squamous cell carcinoma of the mastoid. Finally, a total of 793 cases were included in this study.

### Treatment

Standard according to the German ISSHL guideline was a tapered corticosteroid treatment: 250 mg prednisolone intravenous once daily for the first 3 days, 100 mg intravenously on the 4th day, 75 mg orally on the 5th, 50 mg orally on the 6th, and 20 mg orally on the 7th day. During the inclusion period time from 2009 to 2015 there were various treatment combinations of prednisolone according to the valid version of the clinical guideline for ISSHL treatment: hydroxyethyl starch (HAES), acetazolamide, mannitol, or pentoxifylline. Acetazolamide 500 mg was administered orally as a short infusion once daily for 7 days. Over the long inclusion period, the treatment regimen changed partially for patients with ISSHL. Hydroxyethyl starch (HAES) 6% 250 ml was administered intravenously once daily for 7 days until 2013. Mannitol 15% 250 ml was used intravenously once daily for 3 days and pentoxifylline 300 mg was administered intravenously once daily for 7 days until 2009. The experimental additional NAC treatment was given orally 600 mg two times per day for 7 days. In the absence of improvement or worsening after therapy within 4 weeks, salvage surgery in the form of an intratympanic dexamethasone therapy was offered to the patient. After tympanotomy, 4 mg dexamethasone in GELASPON^®^ (HEYL, Berlin, Germany) was placed into the round window niche at the latest 2 weeks later.

### Hearing classification

The sudden ISSHL or hearing gain (HG) was specified using either 6-tone pure-tone-audiometry (PTA) (0.25 kHz; 0.5 kHz; 1 kHz; 2 kHz; 4 kHz; 6 kHz) or 10-tone PTA (0.125 kHz; 0.25 kHz; 0.5 kHz; 1 kHz; 1.5 kHz; 2 kHz; 3 kHz; 4 kHz; 6 kHz; 8 kHz) [15].

For diseased opposite ear classification, the mean values were calculated from the audiograms of the opposite ear. If the mean was 20 dB ISSHL or more, the ear was classified as diseased. For the calculation of the HG, the cases, each for the 6-tone PTA and the 10-tone PTA, were divided dichotomously about the median of the absolute HG. For evaluation of the outcome, the hearing improvement in Siegel classification and Japan classification was calculated additionally [16, 17]. For the calculations on the influence of ISSHL type, the classes were divided into ISSHL with low-frequency involvement (low frequency ISSHL and low/mid-frequency ISSHL), ISSHL with high-frequency involvement (high frequency ISSHL and high/mid-frequency ISSHL), pantonal ISSHL, and deafness [18]. The median (4 days) was chosen as the time interval for the calculations of the interval from ISSHL event to inpatient treatment initiation for the binary calculations. Shifting the interval to 2, 6, 8, and 14 days did not change the results.

### Statistical analysis

Descriptive analyses were performed using SPSS^®^ Statistics (IBM SPSS Statistics for Windows, Version 23, Armonk, NY, USA). Biometric, anamnestic, audiometric and therapeutic data were collected in a standardized way and selected parameters were dichotomized in a SPSS database. Significance tests were performed using the chi-square test or Fisher’s exact test. Subsequently, selected parameters were examined with regard to their influence on hearing recovery in a univariate analysis. Significant factors from the univariate analysis were included into multivariable regression models to identify independent risk factors for HG, respectively. Both 6-tone PTA (pure tone audiometry) and 10-tone PTA were used to evaluate the hearing findings. Absolute HG, Siegel and Japan classification were used as criteria for evaluation of recovery. Some parameters showed a very strong association with the results in the univariate analyses, the multivariable analyses were repeated again in a further modulation, excluding very strong influencing factors with p < 0.001, in order to identify other independent influencing factors. The significance level of p = 0.05 was set.

## Results

### Patient’s characteristics, treatment characteristics and hearing characteristics

The distribution of patient characteristics, treatment characteristics, and hearing characteristics is shown in Tables 1 and 2. The median age at diagnosis was 60 years and half of patients were women (404 women, 50.9%). Most patients had a pantonal ISSHL (24.8%), followed by low frequency ISSHL (21.1%) and deafness (18%). Nearly all patients received prednisolone (97.7%), in various combinations. A combination with NAC was given to 83.6% of the patients. Slightly more than half of patients started the treatment < 4 days after onset (54.4%), and slightly less than half of the patients had a pre-existing diseased opposite ear. The majority of patients was treated additionally with NAC (83.6%). Mean initial ISSHL in 6-tone PTA and 10-tone PTA was 53.8 ± 34.9 dB and 54.8 ± 34.5 dB, respectively. Mean HG in 6-tone PTA and 10-tone PTA after treatment was 15.5 ± 21.7 dB and 15.2 ± 21.2 dB, respectively. According to the Japanese classification, most of the patients were assigned to type IV (39.8%). One third of the patients was assigned to type I (33.2%), 15.9% of the patients were assigned to type III and 11.1% to type II.

**Table 1:** Patients’ characteristics

Parameter	Absolute (N)	Relative (%)
All	793	100
Gender		
Male	389	49.1
Female	404	50.9
First sudden sensorineural hearing loss		
Yes	547	69.0
Recurrence	246	31.0
Diagnosis		
Hearing loss	655	82.6
Deafness	103	13.0
Vestibulocochlear lesion	31	3.9
Tinnitus	4	0.5
Diseased opposite ear		
Yes	340	42.9
No	453	57.1
Vertigo		
Yes	220	27.7
No	573	72.3
Tinnitus		
Yes	560	70.6
No	233	29.4
Comorbidities		
Yes	726	91.6
No	67	8.4
Vascular risk		
Yes	461	58.1
No	332	41.9
Metabolic syndrome		
Yes	86	10.8
No	707	89.2
Thyroid disease		
Yes	177	22.3
No	616	77.7
Neurological or psychiatric disease		
Yes	222	28.0
No	571	72.0
Coronary artery disease		
Yes	102	12.9
No	691	87.1
Diabetes mellitus		
Yes	121	15.3
No	672	84.7
Hypercholesterolemia		
Yes	56	7.1
No	737	92.9
Hypertension		
Yes	425	53.6
No	368	46.4
Smoking		
Yes	138	17.4
No	655	82.6
Charlson Comorbidity Index (CCI)		
CCI ≥ 1	306	38.6
CCI = 0	487	61.4
	Mean ± SD	Median, Range
Age (years)	57.9 ± 15.7	60, 5–893
Duration until onset of treatment (days)	9.5 ± 19.4	4, 0–247
Duration of treatment (days)	6.7 ± 1.1	7, 3–14

*SD* standard deviation

**Table 2:** Treatment characteristics and hearing characteristics

Parameter	Absolute(N)	Relative (%)
All	793	100
Severity of hearing loss		
Pantonal	197	24.8
Deafness	143	18.0
Low frequency	167	21.1
Low/mid frequency	33	4.2
High frequency	107	13.5
High/mid frequency	88	11.1
No significant hearing loss	9	1.1
Other combinations	49	6.2
Severity of hearing loss II		
Mild-moderate	493	65.9
Severe	255	34.1
Outpatient treatment		
Yes	330	41.6
No	463	58.4
Start of treatment		
< 4 days after onset	431	54.4
> 4 days after onset	362	45.6
Treatment/combinations		
Prednisolone, HAES, NAC	362	45.6
Prednisolone, acetazolamide, mannitol, NAC	170	21.4
Prednisolone, NAC	121	15.3
Prednisolone, acetazolamide, mannitol	34	4.3
Prednisolone, HAES	36	4.5
Prednisolone, pentoxifylline	52	6.6
Other treatment	15	1.9
No treatment	3	0.4
Additional NAC treatment		
Yes	663	83.6
No	130	16.4
Salvage surgery		
Yes	168	21.2
No	625	78.8
Hearing loss	Mean ± SD	Median
Hearing loss 6-tone-PTA	53.8 ± 34.9 dB	44.1 dB
Hearing loss 10-tone -PTA	54.8 ± 34.5 dB	46.5 dB
Hearing gain	27.2 ± 37.2 dB	20.0 dB
Absolute hearing gain 6-tone-PTA	15.5 ± 21.7 dB	7.5 dB
Absolute hearing gain 10-tone-PTA	15.2 ± 21.2 dB	7.0 dB

Recovery classification	I	II	III	IV
	N, (%)	N, (%)	N, (%)	N, (%)
Siegel classification	322, 40.6	79, 10.0	71, 9.0	321, 40.5
Japan classification	263, 33.2	88, 11.1	126, 15.9	316, 39.8

*PTA* pure tone audiometry, *dB* decibel, *NAC* *N*-acetylcysteine, *HAES* hydroxyethyl starch

### Univariable analysis

The results of univariate analyses are shown in Table 3. Patients with ISSHL treated with NAC in addition to prednisolone were close to those without NAC administration regarding the median of absolute HG. The tendency for NAC treatment to perform better, but not significantly, continued in the Siegel classification (frequency independent) and in the 6-tone PTA of the Japan classification. Looking at the Japan classification in 10-tone PTA, NAC treatment was a significant factor of hearing recovery (p = 0.027). No combination of prednisolone with another drugs than NAC had no significant influence on the hearing recovery (all p > 0.05). Japan classification I/II was assigned to 46.5% (308) of patients treated with NAC, whereas only 26.9% (35) of patients who did not receive NAC treatment were assigned as Japan classification I/II. Age, comorbidities, diseased opposite ear and pantonal ISSHL (in Japan classification) were very strong influencing factors (all p < 0.001). Permanent diseases such as hypertension, diabetes mellitus, coronary artery disease (CAD), or vascular risk were significant factors regarding a negative prognosis of hearing recovery in Siegel and Japan classification (all p < 0.05).

**Table 3:** Univariate analyses of association of patient’s characteristics, treatment characteristics and hearing characteristics on absolute hearing gain, and recovery due to Siegel and Japan classification

Parameter	absolute hearing gain (> median)						Siegel classification						Japan classification					
	6-tone-PTA			10-tone-PTA			6-tone-PTA			10-tone-PTA			6-tone-PTA			10-tone-PTA		
	> median	< median		> median	< median		I/II	III/IV		I/II	III/IV		I/II	III/IV		I/II	III/IV	
	N	N	p	N	N	p	N	N	p	N	N	p	N	N	p	N	N	p
Gender			0.435			1.000			**0.039**			**0.023**			0.086			0.152
Male	200	189		211	178		182	207		171	218		160	229		158	231	
Female	196	208		219	185		219	185		212	192		191	213		185	219	
Age			0.943			1.000			** < 0.001**			** < 0.001**			** < 0.001**			** < 0.001**
≤ Median	197	199		215	181		255	141		248	148		232	164		224	172	
> Median	199	198		214	182		146	251		134	263		119	277		119	278	
First event			0.443			0.191			0.592			0.702			0.395			0.757
Yes	268	279		288	259		273	274		261	286		248	299		239	308	
Recurrence	128	118		142	104		128	118		121	125		103	143		103	143	
Charlson Comorbidity Index			0.510			0.510			** < 0.001**			** < 0.001**			** < 0.001**			** < 0.001**
≥ 1	146	160		161	145		129	177		119	187		110	196		107	199	
= 0	250	237		269	218		272	215		263	224		241	246		236	251	
Salvage surgery			0.118			0.281			** < 0.001**			** < 0.001**			0.054			0.096
Yes	93	75		90	78		42	126		38	130		63	105		63	105	
No	303	322		291	334		359	266		344	281		288	337		280	345	
Metabolic syndrome			1.00			1.00			0.361			0.170			0.067			**0.021**
Yes	47	39		47	39		39	47		35	51		30	56		27	59	
No	349	358		383	324		362	345		347	360		321	386		316	391	
Vascular risk			0.886			0.219			** < 0.001**			** < 0.001**			** < 0.001**			** < 0.001**
Yes	229	232		241	220		201	260		191	270		175	286		168	293	
No	167	165		189	143		200	132		191	141		176	156		175	157	
Thyroid disease			0.088			0.266			0.202			0.202			**0.039**			0.103
Yes	78	99		89	88		82	95		81	96		66	111		67	110	
No	318	298		341	275		319	297		301	315		285	331		276	340	
Neurol./psychiatric disease			0.580			0.874			**0.027**			**0.048**			0.080			0.056
Yes	107	115		119	103		98	124		94	128		87	135		87	135	
No	289	282		311	260		303	268		288	283		264	307		259	312	
Smoking			0.455			0.708			0.092			0.225			0.300			0.257
Yes	73	65		77	61		79	59		73	65		67	71		66	72	
No	323	332		353	302		322	333		309	346		284	371		277	378	
Coronary artery disease			0.750			0.523			** < 0.001**			** < 0.001**			** < 0.001**			** < 0.001**
Yes	49	53		52	50		33	69		32	70		33	69		23	79	
No	347	344		378	313		368	323		350	341		368	323		320	371	
Diabetes mellitus			0.491			0.921			**0.003**			**0.003**			**0.007**			**0.003**
Yes	64	57		65	56		46	75		43	78		40	81		37	84	
No	332	340		365	307		355	317		339	333		311	361		306	366	
Hypertension			0.831			0.253			** < 0.001**			**0.001**			** < 0.001**			** < 0.001**
Yes	214	211		222	203		189	236		181	339		161	264		158	267	
No	182	182		208	160		212	156		201	167		190	178		185	183	
Diseased opposite ear			0.430			1.000			** < 0.001**			** < 0.001**			** < 0.001**			** < 0.001**
Yes	164	176		184	156		101	239		86	254		87	253		88	252	
No	232	221		246	207		300	153		296	157		264	189		255	189	
Low frequency			0.072			0.060			** < 0.001**			** < 0.001**			0.100			0.137
Yes	111	89		120	80		127	73		129	71		99	101		96	104	
No	285	308		310	283		274	319		253	340		252	341		247	319	
High frequency			**0.001**			** < 0.001**			** < 0.001**			**0.003**			0.115			0.678
Yes	70	125		85	110		122	73		112	83		96	99		87	108	
No	326	272		345	253		279	319		270	328		255	343		256	342	
Pantonal			**0.007**			**0.048**			**0.017**			**0.004**			** < 0.001**			** < 0.001**
Yes	115	82		119	78		85	112		77	120		59	138		61	136	
No	281	315		311	285		316	280		305	291		292	304		282	314	
Deafness			**0.021**			0.138			** < 0.001**			** < 0.001**			0.926			0.577
Yes 143	84	59		86	57		30	113		27	116		66	77		65	78	
No 650	312	338		344	306		371	279		355	295		287	363		278	372	
Hearing loss			** < 0.001**			** < 0.001**			** < 0.001**			** < 0.001**			1.00			0.753
Mild-moderate (15–59.9 dB)	226	267		251	242		281	212		269	224		202	291		195	298	
Severe (60–130 dB)	164	91		171	84		76	179		69	186		105	150		104	151	
Treatment			** < 0.001**			** < 0.001**			0.776			0.476			0.315			0.073
≤ 4 days after onset	244	187		266	165		220	211		213	218		198	233		199	232	
> 4 day after onsets	152	210		164	198		181	181		169	193		153	209		144	218	
*N*-Acetylcysteine treatment			0.917			0.466			0.349			0.533			0.115			**0.027**
Yes	344	319		376	287		352	311		334	329		213	450		308	355	
No	52	78		54	76		49	81		48	82		39	91		35	95	

Significant* p*-values (*p* < 0.05) in bold

*PTA* pure tone audiometry

### Multivariable analysis

In multivariable logistic regression analysis, all variables which were significant in univariate analysis besides NAC treatment were included (Table 4). The multivariable analyses were repeated again in a further modulation as model 2 (Table 5) and model 3 (Table 6) after exclusion of excluding very strong influencing factors like diseased opposite ear and age (both p < 0.001).The odds ratio (OR) and 95% confidence interval (CI) for worse outcome using the Japan classification in 10-tone PTA are shown in Tables 4, 5 and 6. Prednisolone treatment alone without NAC had a 1.8-fold-increased OR than with additional NAC treatment (OR 1.862; 95% CI 1.200–2.887; p = 0.005) for Japan classification (I/II) in 10-tone PTA. Increased age (OR 1.648; 95% CI 1.139–2.385; p = 0.008), diseased opposite ear (OR 3.048; 95% CI 2.157–4.310; p < 0.001), and pantonal ISSHL (OR 1.891; 95% CI 1.309–2.7320; p = 0.001) were significant factors regarding a negative prognosis of hearing recovery. After excluding very strong influencing factors with p < 0.001 (diseased opposite), CAD showed a 1.8-fold-increased odds ratio than patients without CAD (OR 1.842; 95% CI 1.050–3.231; p = 0.033). Prednisolone treatment alone without NAC treatment showed a 1.7-fold-increased odds ratio than with additional NAC treatment (OR 1.655; 95% CI 1.079–2.538; p = 0.021).

**Table 4:** Multivariable analyses on hearing outcome according to Japan classification (I/II vs. III/IV) model 1 for worse outcome

Parameter	10-tone-PTA			
	OR	Lower 95% CI	Upper 95% CI	p
Age				
≤ Median	1	Reference		
> Median	1.648	1.139	2.385	**0.008**
Opposite ear				
≤ 20 dB	1	Reference		
> 20 dB	3.049	2.157	4.310	** < 0.001**
Charlson Comorbidity Index (CCI)				
CCI ≥ 1	1	Reference		
CCI = 0	1.140	0.771	1.685	0.512
Vascular risk				
No	1	Reference		
Yes	1.572	0.764	3.235	0.219
Coronary artery disease				
No	1	Reference		
Yes	1.681	0.946	2.988	0.077
Diabetes mellitus				
No	1	Reference		
Yes	1.230	0.674	2.245	0.499
Hypertension				
Yes	1	Reference		
No	1.446	0.711	2.941	0.308
Metabolic syndrome				
Yes	1	Reference		
No	1.175	0.598	2.307	0.640
Pantonal hearing loss				
No	1	Reference		
Yes	1.891	1.309	2.732	**0.001**
Additional NAC treatment				
Yes	1	Reference		
No	1.862	1.200	2.887	**0.005**

Significant* p*-values (*p* < 0.05) in bold

*OR* odds ratio, *CI* confidence interval, *NAC* *N*-acetylcysteine

**Table 5:** Multivariable analyses on hearing outcome according to Japan classification (I/II vs. III/IV) model 2 for worse outcome

Parameter	10-tone-PTA			
	OR	Lower 95% CI	Upper 95% CI	p
Age				
≤ Median	1	Reference		
> Median	2.464	1.755	3.459	** < 0.001**
Charlson Comorbidity Index (CCI)				
CCI ≥ 1	1	Reference		
CCI = 0	1.028	0.705	1.500	0.885
Vascular risk				
No	1	Reference		
Yes	1.582	0.784	3.195	0.201
Coronary artery disease				
No	1	Reference		
Yes	1.842	1.050	3.231	**0.033**
Diabetes mellitus				
No	1	Reference		
Yes	1.259	0.701	2.262	0.441
Hypertension				
Yes	1	Reference		
No	1.457	0.729	2.909	0.286
Metabolic syndrome				
Yes	1	Reference		
No	1.036	0.538	1.994	0.917
Pantonal hearing loss				
No	1	Reference		
Yes	1.827	1.276	2.616	**0.001**
Additional NAC treatment				
Yes	1	Reference		
No	1.655	1.079	2.538	**0.021**

Significant* p*-values (*p* < 0.05) in bold

*OR* odds ratio, *CI* confidence interval, *NAC* N-acetylcysteine

**Table 6:** Multivariable analyses on hearing outcome according to Japan classification (I/II vs. III/IV) model 3 for worse outcome

Parameter	10-tone-PTA			
	OR	Lower 95% CI	Upper 95% CI	p
Charlson Comorbidity Index (CCI)				
CCI = 0	1	Reference		
CCI ≥ 1	1.165	0.811	1.674	0.409
Vascular risk				
No	1	Reference		
Yes	1.559	0.789	3.084	0.202
Coronary artery disease				
No	1	Reference		
Yes	2.110	1.220	3.650	**0.008**
Diabetes mellitus				
No	1	Reference		
Yes	1.319	0.741	2.347	0.347
Hypertension				
Yes	1	Reference		
No	1.061	0.549	2.051	0.861
Metabolic syndrome				
Yes	1	Reference		
No	1.080	0.567	2.054	0.815
Pantonal				
No	1	Reference		
Yes	1.980	1.394	2.813	** < 0.001**
Additional NAC treatment				
Yes	1	Reference		
No	1.745	1.147	2.655	**0.009**

Significant* p*-values (*p* < 0.05) in bold

*OR* odds ratio, *CI* confidence interval, *NAC* N-acetylcysteine

## Discussion

The effect on NAC treatment on patients with ISSHL has been rarely investigated worldwide. Current therapeutic approaches on ISSHL are mainly focused on different forms of application of corticosteroids. The aim of this retrospective study was to evaluate the adding administration of NAC to prednisolone treatment on patients with ISSHL. NAC combined to prednisolone treatment was associated with improved hearing outcome on patients with ISSHL according to Japan classification. In multivariable analysis treatment without NAC had an increased odds ratio than with NAC treatment for Japan classification in 10-tone PTA.

In literature, some studies exist on the therapeutic outcome of NAC in combination with steroid treatment in ISSHL, but the data is limited and inconclusive. Kranzer et al. reported in a review of the convincing otoprotective effect of ACC when used with aminoglycosides [9]. A similar conclusion was reached by Kocygit et al., who investigated the otoprotective effect of NAC during administration of ototoxic amikacin in dialysis-associated peritonitis [19]. Kocygit et al. concluded that NAC mainly protects the higher frequency range. The effect of NAC was significant from the fourth week onward. The otoprotective effect of NAC is also being tested in noise-induced hearing loss by an Italian study. Lorito et al. exposed in their study rats to defined noise. A dose-dependent protection of the cochlea by NAC treatment was found. The rats that received high doses of NAC were better protected [20]. Lin et al. investigated the effect of NAC treatment in noise-induced temporary shift on male workers. The authors concluded that the administration of 1200 mg NAC resulted in a significantly reduced “temporary threshold shift” [13].

However, in a randomized, prospective, double-blind, placebo-controlled study from 2015, no benefit of NAC in noice-induced hearing loss was found. For this, soldiers with noice-induced hearing loss were divided into an NAC group (2700 mg per day, starting before a shooting exercise) and a placebo group. After the exercise, their hearing was assessed. In contrast to the post-hoc analysis, there was no advantage for the NAC group when the study was evaluated [12]. Chen et al. investigated the effect of NAC on hearing loss from sudden deafness confined to the inner ear [21]. For this purpose, 35 patients with sudden deafness of unclear origin were treated with NAC 600 mg two times per day for two days and were then discharged with a 3-month consecutive medication, while the control group received a combination treatment of corticosteroid (1 mg/kg), dextran and ginkgo. The group treated with NAC had a significantly greater mean hearing gain than the comparison group with combination treatment (NAC treatment: 43 ± 27 dB vs. combination treatment: 21 ± 28 dB) [21]. Angeli et al. also reported an improvement in hearing recovery of patients with ISSHL with the addition of oral NAC to corticosteroid treatment compared to single therapy without NAC. NAC treatment was given at a dose orally 1200 mg three times daily for two weeks. After 6 months, the NAC group showed an average improvement of 26.1 dB in pure-tone threshold at 500–400 Hz compared to 15.1 dB in single therapy group [22]. In addition, Bai et al. investigated the efficacy of a combination treatment of oral NAC and intratympanic dexamethasone in patients with ISSHL. NAC treatment was given orally 600 mg two times daily for two weeks. There was no improvement in average hearing gain in pure tone audiometry, but a significant hearing gain at 8000 Hz in the NAC group was evident [10]. Chen et al. also reported a significant improvement at 8000 Hz between the NAC group and the non-NAC group. NAC treatment was given orally 600 mg two times daily for at least 1 month. The NAC results were better than the non-NAC group in mean hearing level gain, speech reception threshold gain and speech discrimination score gain, but these differences were not significant [11].

However, the effect of NAC treatment for patients with ISSHL and even with noise exposure is still ambiguous in literature. Lin et al. [14] reported a significant improvement with NAC, while Kopke et al. [9] found no difference. Chen et al. concluded that a greater hearing gain can be achieved with additional NAC administration [21]. In addition Bai et al. and Chen et al. reported of significant improvement at 8000 Hz of a NAC treatment, which is consistent with our findings of improvement in hearing recovery with NAC treatment. But to our knowledge a direct comparison with our study is difficult due to difference of treatment, treatment duration and different classification of hearing recovery.

The present study has due to his retrospective design some limitations. The retrospective design cannot guarantee sufficient information and standardized treatment decision. Causal connections are only traceable to a limited extent. The results from our study showed that the addition of NAC has an impact on the hearing recovery for patients with ISSHL For a better understanding of the role of NAC in treatment of ISSHL, clinical studies for the in a prospective design are needed to provide an adequate evidence.

## Conclusions

This retrospective monocentric study investing the effect of adding NAC to prednisolone treatment on 793 patients with idiopathic sudden sensorineural hearing loss (ISSHL) according to absolute hearing gain, Siegel and Japan classification between 2009 and 2015. In summary, significant factors regarding a negative prognosis of hearing recovery were higher age, diseased opposite (> 20 dB), pantonal ISSHL and prednisolone treatment without additional NAC application. Treatment without addition of NAC had an increased odds ratio than with NAC for Japan classification in 10-tone pure tone audiometry. The results from our study showed that NAC has an important impact on hearing recovery on patients with ISSHL. However, the results of the positive effects of NAC on hearing recovery need to be verified by further analyses in a prospective study.
